# VAR2CSA Signatures of High *Plasmodium falciparum* Parasitemia in the Placenta

**DOI:** 10.1371/journal.pone.0069753

**Published:** 2013-07-25

**Authors:** Eduard Rovira-Vallbona, Isadora Monteiro, Azucena Bardají, Elisa Serra-Casas, Daniel E. Neafsey, Diana Quelhas, Clarissa Valim, Pedro Alonso, Carlota Dobaño, Jaume Ordi, Clara Menéndez, Alfredo Mayor

**Affiliations:** 1 Barcelona Centre for International Health Research (CRESIB, Hospital Clínic-Universitat de Barcelona), Barcelona, Spain; 2 Centro de Investigação em Saúde de Manhiça (CISM), Maputo, Mozambique; 3 Broad Institute, Cambridge, Massachusetts, United States of America; 4 Department of Immunology and Infectious Diseases, Harvard School of Public Health, Boston, Massachusetts, United States of America; 5 Department of Pathology, Hospital Clínic-Universitat de Barcelona, Barcelona, Spain; Centro de Pesquisa Rene Rachou/Fundação Oswaldo Cruz (Fiocruz-Minas), Brazil

## Abstract

*Plasmodium falciparum* infected erythrocytes (IE) accumulate in the placenta through the interaction between Duffy-binding like (DBL) domains of parasite-encoded ligand VAR2CSA and chondroitin sulphate-A (CSA) receptor. Polymorphisms in these domains, including DBL2X and DBL3X, may affect their antigenicity or CSA-binding affinity, eventually increasing parasitemia and its adverse effects on pregnancy outcomes. A total of 373 DBL2X and 328 DBL3X sequences were obtained from transcripts of 20 placental isolates infecting Mozambican women, resulting in 176 DBL2X and 191 DBL3X unique sequences at the protein level. Sequence alignments were divided in segments containing combinations of correlated polymorphisms and the association of segment sequences with placental parasite density was tested using Bonferroni corrected regression models, taking into consideration the weight of each sequence in the infection. Three DBL2X and three DBL3X segments contained signatures of high parasite density (*P*<0.003) that were highly prevalent in the parasite population (49–91%). Identified regions included a flexible loop that contributes to DBL3X-CSA interaction and two DBL3X motifs with evidence of positive natural selection. Limited antibody responses against signatures of high parasite density among malaria-exposed pregnant women could not explain the increased placental parasitemia. These results suggest that a higher binding efficiency to CSA rather than reduced antigenicity might provide a biological advantage to parasites with high parasite density signatures in VAR2CSA. Sequences contributing to high parasitemia may be critical for the functional characterization of VAR2CSA and the development of tools against placental malaria.

## Introduction

*Plasmodium falciparum* binds carbohydrate molecules to recognize, attach and invade cells both in the human and mosquito hosts [1]. Among the different parasite-encoded ligands involved in these interactions, those containing a specific fold structure called Duffy-binding like (DBL) domain bind to host receptors such as glycophorin A, complement receptor 1 and chondroitin sulphate-A (CSA) to promote the invasion of erythrocytes by merozoites, the binding of infected erythrocytes (IE) to uninfected erythrocytes (‘rosettes’) and the sequestration of mature parasites in the placenta, respectively [2]. Although structural requirements are still incompletely understood, all these interactions are targets for the treatment and/or prevention of the disease, including vaccine development.

**Figure 1:**
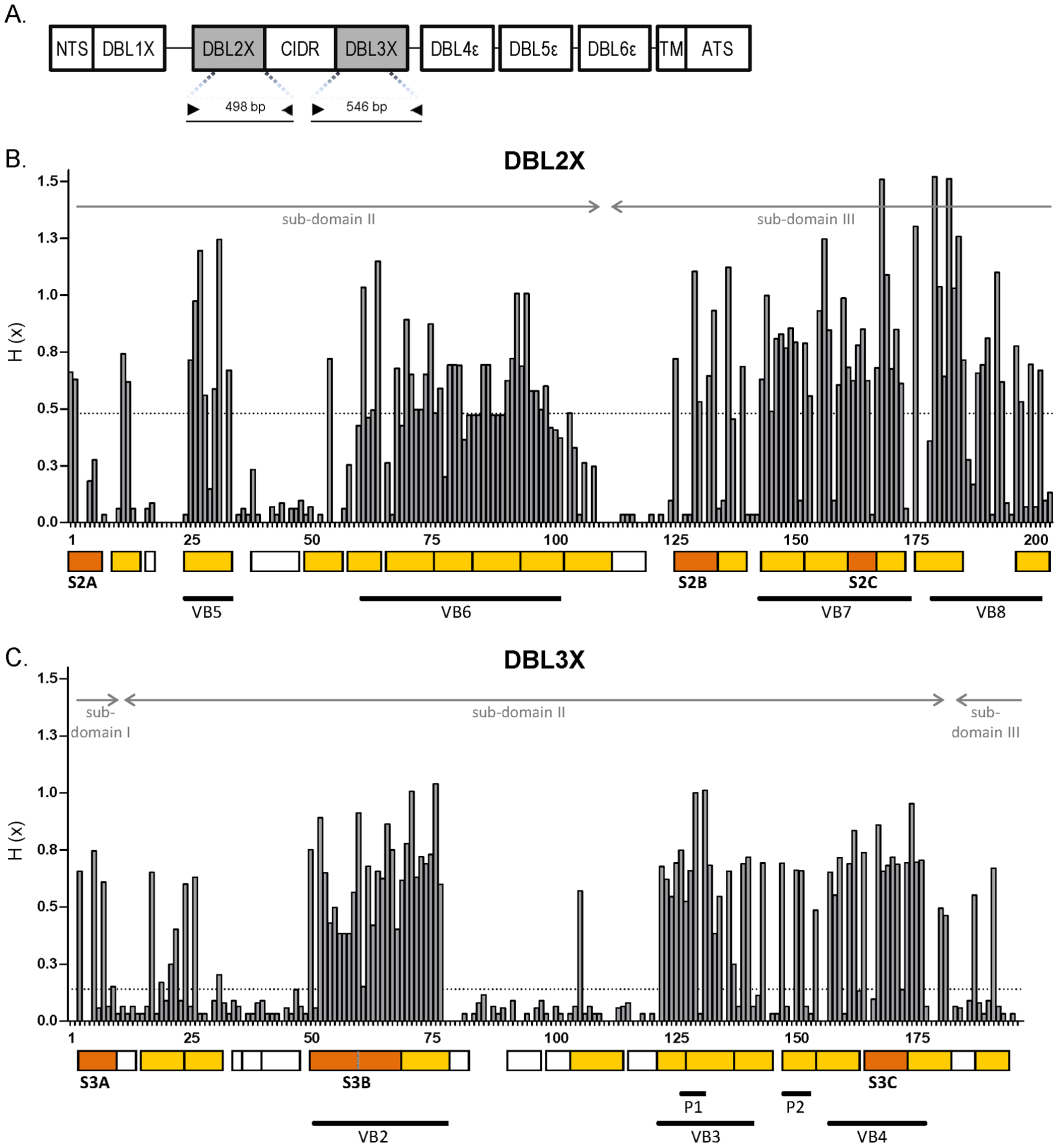
Variability in DBL2X and DBL3X amino acid sequences. *(A)* VAR2CSA domain structure and regions covered by sequencing in the reference strain A4 (NTS: N-terminal segment; DBL, Duffy-binding like; CIDR, cysteine-rich inter-domain region, TM: trans-membrane; ATS: acidic-terminal segment). *(B, C)* Shannon entropy values were calculated on the multiple sequence alignment of 176 DBL2X (and Dataset S1) and 191 DBL3X amino acid sequences (and Dataset S2) transcribed by placental parasites. Dotted horizontal lines indicate median of all positive entropy values (H_DBL2X_ = 0.48 and H_DBL3X_ = 0.14). Arrows indicate DBL sub-domain boundaries [16]. White boxes delimit segments with residues below the entropy threshold; colored boxes delimit variable segments carrying signatures not associated (yellow) or associated with high placental parasite density (orange, Bonferroni corrected Wald test: *P*[S2A] = 0.0020, *P*[S2B, S2C, S3A and S3B]<0.001; *P*[S3C] = 0.0032). Regions corresponding to previously reported parity-linked motifs (P1, P2) [34], [35] and variable blocks (VB) [38] are underlined.

The accumulation of *P. falciparum* IE in the placental intervillous spaces is an important cause of premature delivery and fetal growth restriction in malaria endemic areas, leading to an increased risk of low birth weight (LBW) and mortality in newborns [3], [4], [5]. IE adhesion to CSA is mediated by VAR2CSA, a 350 KDa *P. falciparum* variant antigen codified by the highly polymorphic *var2csa* gene and expressed on IE surface [6], [7]. Antibodies against VAR2CSA are acquired after exposure to *P. falciparum* during pregnancy, increase with parity and have been associated with improved pregnancy outcomes [8], [9], [10], although conclusive evidence of their protective effect remains controversial [11], [12], [13], [14], [15]. Extracellular VAR2CSA is composed of 6 DBL domains plus a cysteine-rich inter-domain region (CIDR) between DBL2X and DBL3X [16] (Figure 1). Although recombinant DBL2X, DBL3X and DBL6ε have shown to individually bind CSA [17], [18], [19], [20], the precise molecular interaction is still not known in detail due to the absence of full-length VAR2CSA crystal structures. Recent studies suggest that residues providing the highest binding specificity lie within DBL2X domain [21], with contribution from DBL1X and DBL3X [22], [23].

The high variability of VAR2CSA has hampered the identification of residues critical for the induction of antibodies blocking adhesion to CSA in a strain-transcending manner [13], [24], [25], [26],[27],[28],[29],[30],[31]. Polymorphisms in VAR2CSA may affect the intensity of IE binding to CSA [32],[33] and contribute to the evasion of host immunity. Characterization of these amino acid changes can help to understand the mechanisms leading to placental sequestration of IE and inform the development of new control tools to reduce the adverse effects associated with placental malaria. Previous studies identified two amino acid motifs in DBL3X that were over-represented in parasites isolated from Senegalese and Kenyan primigravid women (PG) [34],[35], suggesting that parasites with these motifs may bind more efficiently to placenta in women with limited immunity. Polymorphisms in DBL5ε have also been associated with the CSA-binding intensity of placental isolates [36], but a direct role for this domain in CSA-VAR2CSA interaction is unclear [17],[22],[23],[37]. Here, we hypothesized that polymorphisms in VAR2CSA may affect parasite densities in the placenta by altering the antigenicity of domains involved in the adhesion to CSA and their ability to escape immune recognition by the host. To test this, polymorphisms in DBL2X and DBL3X domains were assessed for their association with placental parasite density, and natural immunogenicity of identified sequence variants was evaluated.

## Results

### Characteristics of pregnant women and placental isolates

Median age of the 20 women included in the study was 20 years (interquartile range [IQR] 18–26), and 10 of them (50%) were PG. The median number of pregnancies among multigravid women (MG) was 3 (IQR 2–6). Five newborns presented LBW. Placental parasite density ranged from 208 to 213451 parasites/μl (median of 12192 parasites/μl). There was no significant difference in parasite density between PG (14781 parasites/μl, IQR 4425–39171) and MG (7740 parasites/μl, IQR 2394–67396; *P* = 0.605) or between mothers delivering LBW (15740 parasites/μl [IQR 13821–17598]) and normal weight babies (8199 parasites/μl [IQR 1995–67396]; *P* = 0.327). A median number of 4 different merozoite surface protein (*msp*) genotypes were found in placental isolates (IQR 3–6).

### Sequence diversity of DBL2X and DBL3X

Three-hundred and seventy-three DBL2X and 328 DBL3X sequences were obtained from the RNA of 20 placental isolates after PCR amplification, transformation in *E. coli* and sequencing (Table 1). Among them, 203 DBL2X (54%) and 222 DBL3X (68%) sequences were different at the nucleotide level, with lengths ranging from 450 to 555 nucleotides for DBL2X and from 537 to 591 for DBL3X (Figure 1). Pair-wise nucleotide diversity (π) in the sequenced regions of DBL2X and DBL3X was 0.0729 and 0.0617, respectively. In each isolate, a median of 10 DBL2X and 12 DBL3X different nucleotide sequences were identified, exceeding by >2-fold the number of genotypes estimated by standard PCR-typing of *msp* alleles (Table 1). The most common sequence in each placental infection accounted for 55% (DBL2X) and 42% (DBL3X) of the sequenced clones (Table 1). The number of sequences transcribed by *P. falciparum* isolates was not significantly associated with age, parity or parasite density in the placenta.

**Table 1:** Sequenced clones and DBL2X/DBL3X sequence diversity in 20 placental isolates.

	Median *n* per infection (IQR)		Total	
	DBL2X	DBL3X	DBL2X	DBL3X
Sequenced clones	19 (17–20)	17 (14–19)	373	328
Different nucleotide sequences	10 (7–15)*	12 (9–14)*	203	222
Different amino acid sequences	9 (5–14)*	10 (8–12)*	176	191
Frequency of most common nucleotide sequence	55% (20%–84%)	45% (20%–54%)	*NA*	*NA*
Different nucleotide sequences per *msp* alleles	2.2 (1.5–2.6)	2.5 (1.7–2.8)	*NA*	*NA*

IQR, interquartile range; *msp*, merozoite surface protein; *NA*, not applicable.

* Normalized values (see *Materials and Methods*).

Among all different nucleotide sequences, 176 (87%) from DBL2X and 191 (86%) from DBL3X were unique at the protein level (Table 1) and used for the multiple amino acid sequence alignments (see Datasets S1 and S2). Global mean pair-wise amino acid identity was 73% for DBL2X and 82% for DBL3X. Ignoring gaps, 124 out of the 205 DBL2X alignment positions (61%) and 145 out of the 198 DBL3X positions (73%) were found to be polymorphic. The level of amino acid variability at each site was quantified through the Shannon entropy score (H), yielding median values of 0.48 for DBL2X and 0.14 for DBL3X (Figure 1). Regions with the highest entropy (i.e., scores above the median) corresponded to already defined variable blocks (VB) of VAR2CSA [38]: VB5 (alignment positions [pos.] 26–34; for reference, amino acids 693–670 in the A4 strain, AAQ73926), VB6 (pos. 61–103; 727–748 in A4), VB7 (pos. 145–175; 790–810 in A4) and VB8 (pos. 181–203; 816–832 in A4) in DBL2X, and VB2 (pos. 51–78; 1324–1337 in A4), VB3 (pos. 124–142; 1382–1399 in A4) and VB4 (pos. 159–178; 1417–1435 in A4) in DBL3X.

### Signatures associated with placental parasite density

A segmentation analysis was conducted to divide amino acid sequence alignments in <10-amino acid segments containing combinations of strongly correlated polymorphic positions, independent from the polymorphisms in other segments [38]. A segment was then categorized into a maximum of 5 sequence types according to the pattern of amino acids in the polymorphic positions of the segment. Alignments of DBL2X and DBL3X were partitioned in 21 and 24 segments, respectively (Figure 1). Eighteen DBL2X segments had an entropy score above the median, covering 147 alignment positions (72%) with a median length of 8 residues and a median number of 3 sequence types per segment. The 15 DBL3X segments with high entropy scores covered 129 alignment positions (65%) and had a median of 9 residues and 3 sequence types per segment. Amino acids in positions 51–60 and 61–69 of the DBL3X alignment were segmented in two perfectly linked sequences and were therefore merged as a single segment of 19 residues. The sequence types in all the 18 DBL2X and 14 DBL3X variable segments were analysed for their association with parasite density in the placenta using regression models and Bonferroni correction for multiple comparisons, by setting *P* values for statistical significance at 0.0028 [0.05/18] for DBL2X and 0.0036 [0.05/14] for DBL3X. Three DBL2X segments (S2A, S2B and S2C) and 3 DBL3X segments (S3A, S3B and S3C) contained sequences associated with high parasite density (or high density signatures, HDS) compared to the reference sequence (or low density signature, LDS; Table 2 and Table S1; see Figure 1 for segment positions). Adjusting regression models by parity and age did not change results of the univariate analysis. Placental isolates transcribing *var2csa* genes with HDS in the 3 DBL2X segments or in the 3 DBL3X segments were associated with higher parasite density than isolates transcribing sequences with other combinations of sequence types (proportional increase in placental parasite density for DBL2X: 8.1, 95% CI [3.7–17.9], *P*<0.001; and DBL3X: 4.9, 95% CI [2.0–12.0], *P*<0.001; Wald tests).

**Table 2:** Amino acid segments and sequence types associated with *P. falciparum* density in the placenta.

Segment	Position^a^	Sequence type	Consensus^b^	Mean parasites/μl ×10^3^ (range)	IRR (95% CI)	*P* c	Median B epitope score (IQR)^d^
**DBL2X**							
S2A	668–674	S2A.1	EYTKxLE	59.0 (0.2, 213.4)	4.6 (1.7, 12.5)	0.003	0.1 (−0.3, 0.4)
		S2A.2	DFTK(D/N)xE	12.8 (0.2, 77.1)	*ref.*		−0.01 (−0.2, 0.3)
S2B	772–780	S2B.1	EQRQx(K/E)VNA	88.4 (0.2, 213.4)	10.7 (3.4, 33.6)	<0.001	0.2 (−0.1, 0.4)
		S2B.2	KQRQENVNA	58.6 (0.3, 134.7)	7.1 (2.6, 19.8)	<0.001	0.2 (0.1, 0.5)
		S2B.3	(E/K)QRQxKVKx	14.0 (0.2, 7.3)	1.7 (0.5, 5.6)	0.383	0.3 (0.2, 0.6)
		S2B.4	(E/K)QRQ(E/A)KVNA	8.2 (0.2, 134.7)	*ref.*		0.1 (0.02, 0.4)
S2C	802–803	S2C.1	(E/K)CKNK(C/−)	47.1 (9,2, 202.9)	27.4 (5.6, 133.7)	<0.001	0.3 (0.3, 0.5)
		S2C.2	(K/E)C	44.9 (0.2, 213.4)	26.1 (8.8, 77.3)	<0.001	0.6 (0.6, 0.7)
		S2C.3	EC(E/K)KKC	1.7 (0.2, 4.9)	*ref.*		0.6 (0.5, 0.6)
**DBL3X**							
S3A	1275–1283	S3A.1	NLWxK(S/R)Y(G/V)	62.8 (0.2, 202.9)	13.8 (4.0, 47.1)	<0.001	0.3 (0.01, 0.7)
		S3A.2	ELWYK(S/R)Y(G/V)	48.7 (0.3, 213.4)	10.7 (3.4, 33.5)	<0.001	0.1 (−0.2, 0.4)
		S3A.3	ELWDKxYG	4.6 (0.2, 39.2)	*ref.*		0.5 (0.2, 0.7)
S3B	1324–1332	S3B.1	xNx(M/−)(D/−)(K/−)K(G/−) (Q/−)(K/−)x(K/−)	48.4 (0.3, 213.4)	12.6 (3.1, 51.5)	<0.001	1.5 (0.8, 1.8)
		S3B.2	RNPMKEGGEDGKG(K/N)QKEGG	3.8 (0.2, 67.3)	*ref.*		1.9 (1.2, 2.1)
S3C	1424–1431	S3C.1	xKKQKKxGT	69.6 (0.5, 213.4)	6.0 (2.1, 17.5)	0.001	1.3 (0.9, 1.4)
		S3C.2	NKKxK(K/E)(N/D)GT	23.0 (0.3, 134.7)	2.0 (0.7, 5.8)	0.214	1.3 (0.8, 1.4)
		S3C.3	xKK(Q/−)(N/D)NK(C/G)T	11.6 (0.2, 77.2)	*ref.*		0.9 (0.8, 1.0)

IRR, incidence rate ratio (i.e., proportional increase in placental parasite density); CI, confidence interval; IQR, interquartile range; *ref.,* reference category.

a Referred to A4 VAR2CSA.

b ‘x’ indicates positions with 3 or more possible residues, including alignment gaps (see Datasets S1 and S2). Conserved lysine in S3B (K1328 in A4 strain) is underlined.

c Negative binomial regression with robust estimation of variance.

d Median of all BepiPred scores for each residue within a sequence type.

Parity was not associated with the transcription of any sequence type in the variable segments obtained through the segmentation method (*P*>0.0045, Wald tests; see results for S2A–C and S3A–C in Table S1). The segment containing a 5-amino acid motif in DBL3X previously associated with parity in infected women from Senegal (region P2 in Figure 1; amino acids 1407–1411 in A4) [34] was not associated with increased parasite density in the regression analysis of the present study (*P* = 0.048, Wald test). A second DBL3X motif of 4 amino acids associated with parity in Kenya (region P1 in Figure 1; amino acids 1385–1389 in A4) [35] was split in two different segments in the present analysis, none of them containing a motif associated with increased placental density (*P* = 0.024 and *P* = 0.008, respectively). Similarly, none of the segments was associated with LBW by logistic regression models (*P*>0.122, Wald tests; see results for S2A–C and S3A–C in Table S1), although women carrying LDS sequence types at S2C or S3B (3 and 5 women, respectively) all delivered babies with normal weight (*P*<0.001, Fisher's exact, Table S1).

### Amino acid changes and evolutionary analysis

Single amino acid substitutions occurred in all the six identified segments, with deletions being present in S2C, S3B and S3C. Substitution matrices [39] were used to assess the potential impact on DBL structure/function of amino acid exchanges between the most frequent HDS and LDS sequence types (Table S1), whereby positive scores indicate favorable substitutions with low impact and vice versa. A favorable substitution was found in S2A (F_LDS_-Y_HDS_, score +2), whereas unfavorable exchanges were present in S3B (G_LDS_-K_HDS,_ −1) and S3C (C_LDS_-G_HDS_, −6) (Table S1). A third unfavorable substitution found in S3A (D_LDS_-Y_HDS_, −6) seems unlikely to have an effect on parasite density since this exchange can also occur between two HDS sequences (D_HDS_-Y_HDS_).

The effect of natural selection on each segment was evaluated by estimating the difference in the numbers of synonymous and non-synonymous nucleotide substitutions per site (dS and dN, respectively) as well as Tajima's D and Fu and Li's D statistic [40]. Mean dN-dS differences were −1.058 for S2A, 0.197 for S2B, −1.294 for S2C, 1.819 for S3A, 0.704 for S3B and 2.583 for S3C, with positive values being indicative of natural selection that promotes polymorphisms at non-synonymous sites. Comparison of dN and dS using a codon-based test (Nei-Gojobori method for Z-test of selection) showed a significant departure from neutrality for segments S3A (*P* = 0.036) and S3C (*P* = 0.006). Fu and Li's D was significant for S3A (−3.495, *P*<0.020) and S3C (−2.705, *P*<0.005) together with moderate negative Tajima's D values (Figure S1), suggesting an excess of rare variants compared to expectation under a neutral model of evolution.

### Surface exposure and epitope prediction

**Figure 2:**
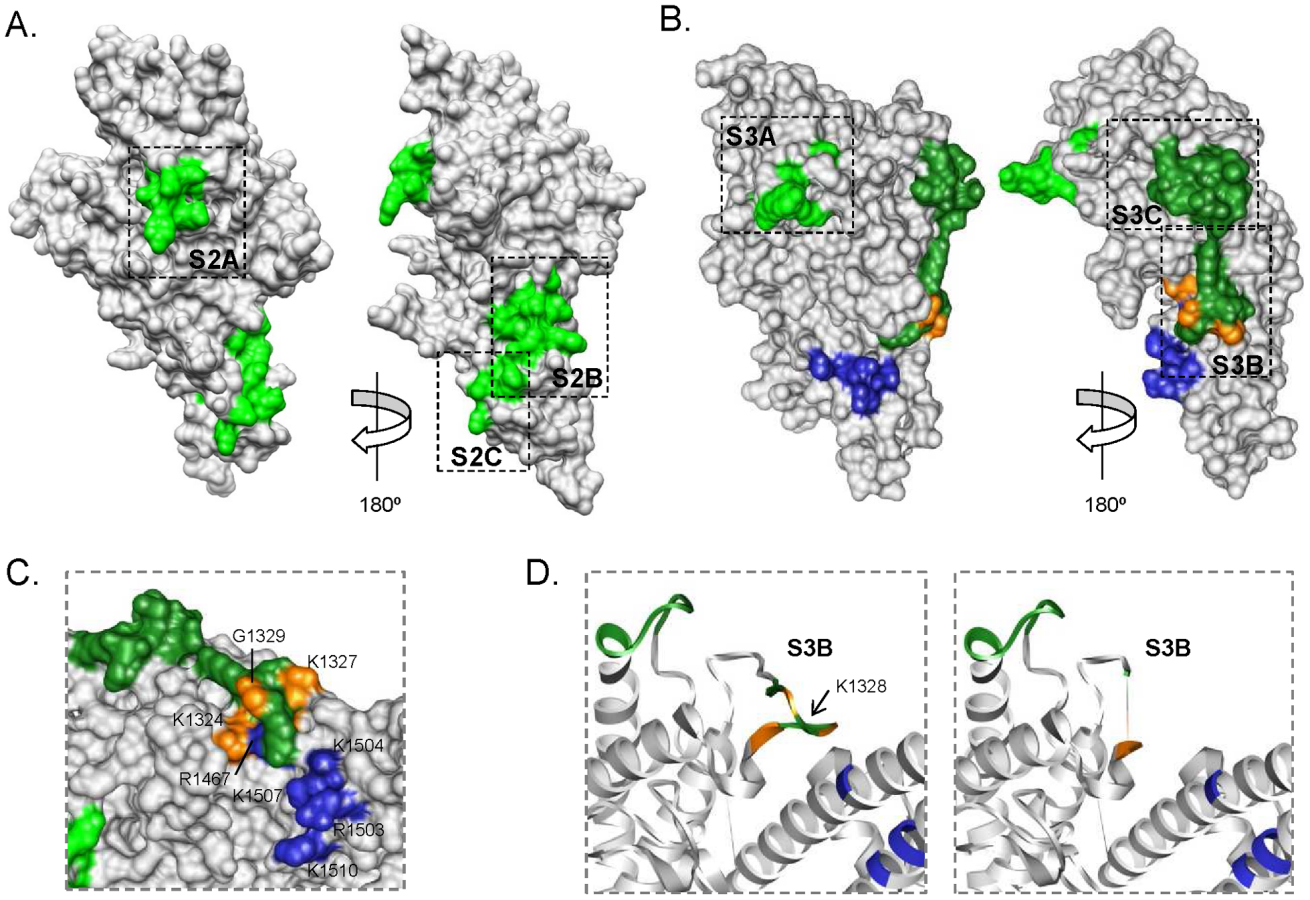
Surface mapping of segments containing high parasite density signatures. (*A*) 3D model for DBL2X domain. (*B*) Crystal structure of DBL3X domain (PDB 3BQK). Segments with BepiPred scores ≥0.9 are shown in dark grey. Residues predicted to interact with CSA sulfate groups by Higgins [19] and Singh *et al.* [18] are colored in blue (R1467, R1503, K1504, K1507, K1510), or orange for those that are also part of the S3B segment (K1324, K1327, G1329). (*C*) Detail of predicted CSA binding amino acids [18],[19]. *(D)* Ribbon visualization of the DBL3X loop containing segment S3B and K1328 in the presence (PDB 3BQK, left) or absence (PDB 3BQI, right) of sulfate groups [19].

Mapping the 6 amino acid segments containing sequences associated with high parasite density on 3D structures of individual domains showed that they were all localized in exposed areas on the surface of the proteins (Figure 2). Segments S3B and S3C in DBL3X were assigned as predicted B cell epitopes according to existing database records with a specificity of 91% in BepiPred (Table 2 and Figure 2). BepiPred scores between HDS and LDS motifs were similar in all segments.

### Antibody recognition of amino acid sequences associated with high parasite density

**Figure 3:**
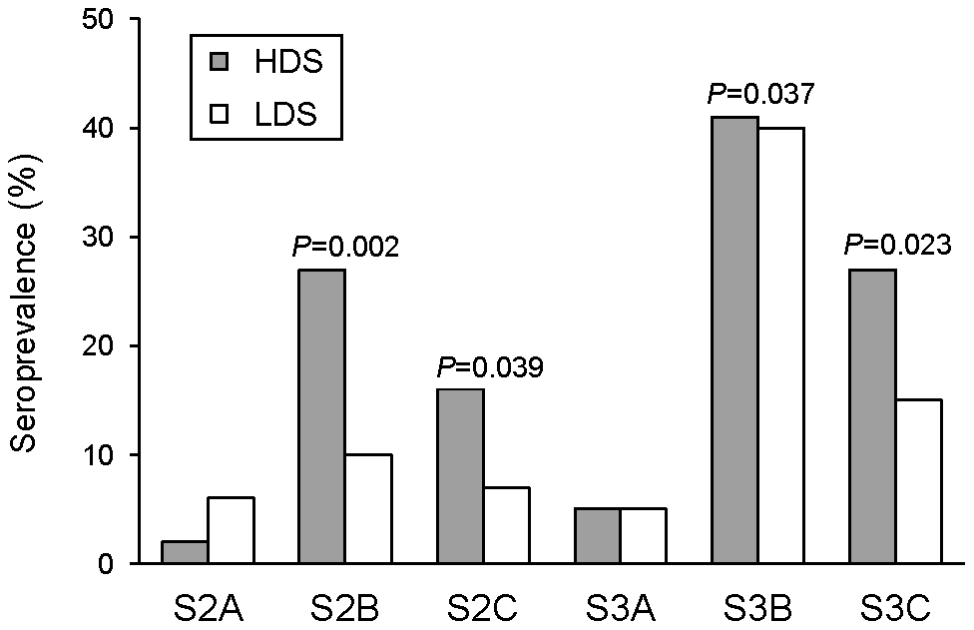
Recognition of DBL2X and DBL3X peptides by plasma IgG from 100 pregnant women. Seroprevalence is represented by bars as the % of responders. Peptides representative of signatures of high (HDS) and low (LDS) parasite density (Table 2 and *Materials and Methods*) and their optical density thresholds for seroprevalence (mean plus 3 standard deviations of OD in negative controls) were: HDS, S2A.1 = 0.690, S2B.1 = 0.210, S2C.1 = 0.266, S3A.1 = 0.293, S3B.1 = 0.140, S3C.1 = 0.295; LDS, S2A.2 = 0.450, S2B.4 = 0.266, S2C.3 = 0.480, S3A.3 = 0.366, S3B.2 = 0.151, S3C.3 = 0.195. *P*-values were calculated using McNemar's test (tests for S2A and S3A were not applicable due to low seroprevalence).

Linear peptides representative of HDS and LDS sequences in each segment were tested for IgG recognition by 100 plasmas from Mozambican pregnant women at delivery (41 from PG and 59 from MG) to evaluate differences in natural immunogenicity. S2B_HDS_, S3B_LDS/HDS_ and S3C_HDS_ peptides were recognized by >25% of the plasmas tested (Figure 3). In contrast, recognition of peptides for S2A and S3A was low (<6%). HDS peptides from S2B, S2C and S3C were significantly more recognized by plasmas than LDS peptides (McNemar's test for paired data, Figure 3). A similar trend was observed for S3B, although the marked difference in peptide lengths between S3B_HDS_ (7 amino acids) and S3B_LDS_ (19 amino acids) may confound differences in immunogenicity for this specific comparison.

### Geographical distribution of high parasite density signatures

**Figure 4:**
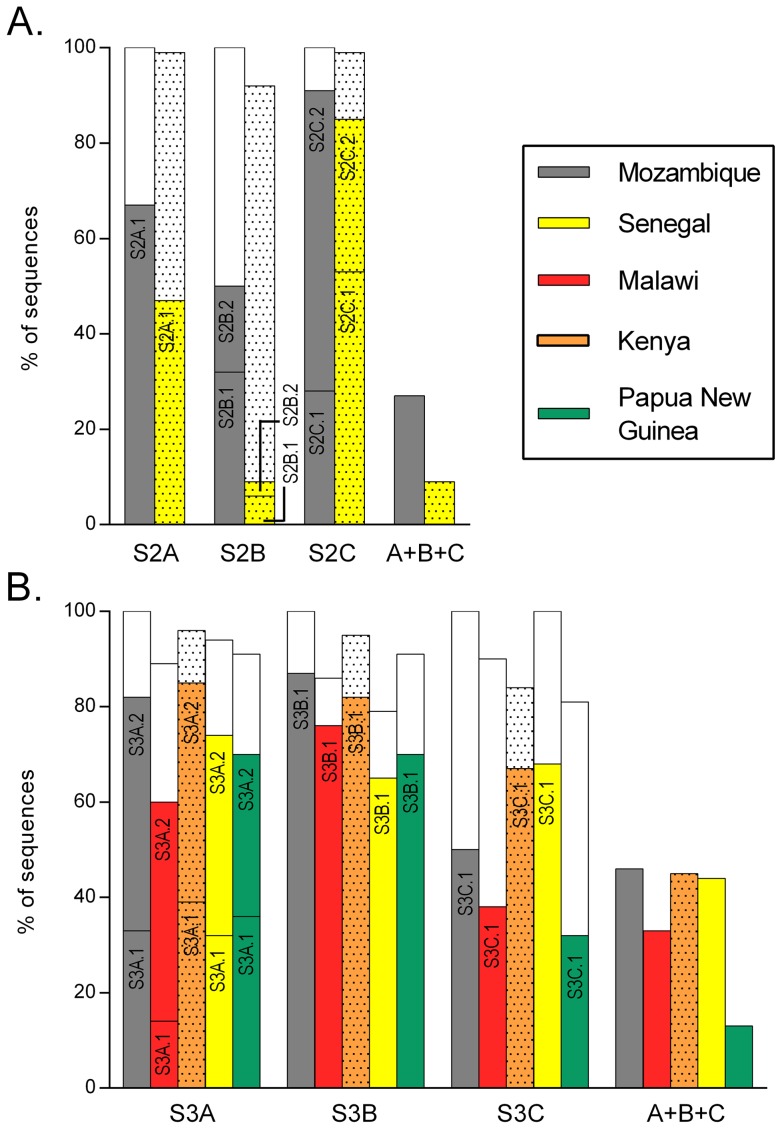
VAR2CSA sequences with high parasite density signatures in different *P. falciparum* populations. Colored bars show the frequency of the HDS sequence types identified among Mozambican isolates (Table 2) in DBL2X *(A)* and DBL3X *(B)* sequence sets from other geographical origins. Likewise, white bars show the frequency of LDS sequences. Plain columns indicate cDNA sequences, dotted columns indicate gDNA sequences. Columns ‘A+B+C’ show prevalence of VAR2CSA sequences with 3 signatures of high parasite density.

The frequency at which HDS were found among the repertoire of VAR2CSA variants expressed by Mozambican isolates was estimated from the 176 DBL2X and 191 DBL3X VAR2CSA unique amino acid sequences (Figure 4). At least one HDS was present in 163 (93%) DBL2X and 187 (98%) DBL3X sequences obtained in this study, with 47 (27%) and 88 (46%) of DBL2X and DBL3X domains, respectively, carrying the 3 HDS sequence types. Presence of motifs associated with high placental parasite density in publicly available DBL2X and DBL3X sequences from other geographical areas was evaluated. Sequences from all HDS signatures identified in Mozambique were found in the DBL2X sequences from Senegal [41] and in the DBL3X sequences from Senegal, Malawi, Kenya and Papua New Guinea parasites at similar prevalence [34],[35],[42],[43], ranging from 9% (n = 3 out of 34, S2B_HDS_ in Senegal) to 85% (n = 64 out of 76, S3A_HDS_ in Kenya). Sequences containing 3 HDS motifs accounted for 9–45% of the non-Mozambican sets of sequences.

## Discussion

Development of interventions against malaria targeting *P. falciparum* cytoadhesion, such as vaccines or anti-adhesive adjuvant therapies, is hampered by our limited understanding of the effect that the extreme polymorphism of parasite ligands may have on host-parasite interactions. This study identifies 6 amino acid motifs in DBL2X and DBL3X domains of VAR2CSA -the ligand mediating *P. falciparum* IE adhesion to human receptor CSA- that are predominantly transcribed by parasites causing placental infections of high parasite density. Four out of the 6 segments have B cell epitope features (including exposure on the surface of the domain and high B epitope scores by *in silico* predictions, as well as significant recognition by IgGs from malaria exposed pregnant women), suggesting they are targets of naturally acquired immunity. Moreover, the high prevalence of signatures associated with increased parasite density among placental isolates from Mozambican and other geographical areas suggest they may be relevant to the breadth of maternal antibody responses against diverse geographical isolates [8].

Placental isolates analysed in this study showed a high degree of genetic complexity, reaching a median of 10–12 different *var2csa* sequences per infection, that exceeds by 2-fold the number of *msp* genotypes [35],[44]. This ratio supports previous reports showing multiple *var2csa* copies in parasite genomes [41],[45],[46], what may provide the parasite means to escape immune response throughout pregnancy [46]. The degree of polymorphism of DBL2X (π = 0.062) and DBL3X (π = 0.073) sequences expressed by Mozambican parasites is similar to the level described in smaller sequence sets from other geographical areas [34],[35],[43], indicating that diversity is limited despite still being considerably higher than for other polymorphic malarial antigens such as the apical membrane antigen-1 (π = 0.016) [47].

The evolutionary tests used for detecting departure from neutrality showed evidence that polymorphisms observed in 2 DBL3X segments (S3A and S3C) were maintained by natural selection. Codon-based test comparing dN and dS rates suggested that diversifying selection is acting on these regions with non-synonymous variants being positively selected [48]. Moreover, this was supported by negative Fu and Li's D indicating an excess of rare polymorphisms. Although other factors such as recent population expansion may also contribute to the shift towards a low-frequency spectrum of polymorphisms and despite the limited sample size to conduct more robust evolutionary analysis, the consistency with dN-dS data suggests that signatures of positive selection may promote adaptation of VAR2CSA to optimize placental colonization.

Amino acid polymorphisms in DBL2X and DBL3X may affect the folding and tertiary structure of the domains, leading to different binding affinities to CSA or changing the degree of immune recognition by antibodies naturally acquired during pregnancy, as previously suggested for the *P. vivax* Duffy-binding protein [49]. Results of this study point towards the relevance of polymorphisms in the S3B segment of DBL3X, a flexible loop of 19-amino acids with a conserved lysine residue (K1328 in A4 strain; see Figure 2). This lysine stabilizes the loop conformation in the presence of CSA and facilitates the establishment of hydrogen bonds between residues surrounding lysine and the receptor sulfate groups [18],[19]. It has been hypothesized that, when the protein is unbound to the receptor, the polymorphism and flexibility of the S3B loop protects conserved features of the CSA-binding pocket from immune recognition [18],[19]. In accordance with this, S3B matched databases of B cell epitopes with high specificity and both S3B_HDS_ and S3B_LDS_ peptides were recognized by 40% of plasmas from pregnant women (Figure 3). S3B segment shows a marked dimorphic pattern, with signatures of high parasitemia being those deleted around K1328 (Table 2). The unfavourable score for the substitution of a lysine (characteristic of S3B_HDS_ motifs) by a glycine (characteristic of S3B_LDS_; G1327K substitution in A4 strain) suggests this may be one of the changes potentially affecting the domain properties. Similar B epitope scores and IgG recognition rates between HDS and LDS sequences does not support a reduced antigenicity for motifs associated to high parasite densities. In accordance with this interpretation, HDS peptides from S3C in DBL3X as well as S2B and S2C in DBL2X were even significantly more recognized than their corresponding LDS, although we cannot exclude that some of the women had not had contact with some of the epitopes tested. These results reinforce the concept that limited host antibody-mediated immunity against sequences associated with high parasite density may not be the mechanism contributing to a high parasite biomass in the placenta. Rather, polymorphisms in HDS sequences such as the unfavorable loss of a cysteine in S3C or the substitution of a glycine by a lysine in S3B may provide an increased affinity of binding to CSA, either through modifications at this specific site or by changes in the conformation of nearby regions. Whether adhesion affinity of parasites expressing different VAR2CSA signatures varies enough to affect placental parasitemia could not be addressed in this study due to the lack of cryopreserved isolates, an issue that will need to be assessed in the future.

In conclusion, the present work shows that the sequence segmentation method [38] applied to genetic association studies provides a useful tool to identify specific polymorphisms of highly variable DBL proteins that may contribute to phenotypic diversity. In particular, we provide evidence of *P. falciparum* parasites expressing specific VAR2CSA variants that have the potential to reach a high parasitemia in the placenta and eventually increase the risk of poor pregnancy outcomes. The motifs in VAR2CSA associated with high placental parasitemia in this study may be of relevance to understand the molecular mechanisms mediating parasite sequestration to host tissues, as well as for the development of new preventive tools against placental malaria.

## Materials and Methods

### Ethics statement

The study protocol was approved by the National Ethics Review Committee of Mozambique and the Hospital Clinic of Barcelona Ethics Review Committee. All samples were collected only after written informed consent was given by the patient.

### Study area, participants and samples

The recruitment of participants and sample collection was carried out between March 2004 and November 2005 at the Manhiça District Hospital (MDH, Manhiça, Mozambique). *P. falciparum* transmission was perennial with some seasonality and the estimated entomological inoculation rate was 38 infective bites/person/year [50]. At the time of the recruitment, intermittent preventive treatment during pregnancy was not yet implemented and malaria control during pregnancy relied exclusively upon case management.

*P. falciparum* placental isolates from 20 women delivering at the MDH were included in this study. After obtaining written informed consent, the placenta was collected and processed at the Centro de Investigação em Saúde de Manhiça (CISM). Several one-centimetre incisions were made in the endometrial side of placentas. Four ml of blood were withdrawn into EDTA tubes and centrifuged. IE were snap frozen in ethanol and dry ice and stored at −80°C. Giemsa-stained thick and thin blood smears were used to calculate parasite density. Briefly, *P. falciparum* asexual stages and leukocytes were counted until 500 parasites or leukocytes were reached, and parasite density was estimated using an assumed leukocyte count of 8000 leucocytes/μl of blood. Two 50 μl blood drops were spotted onto filter paper (Schleicher and Schuell; no. 903TM). Women were treated according to Mozambican national guidelines.

### *msp* genotyping

Parasite genomic DNA (gDNA) was isolated from blood drops onto filter papers (QIAamp DNA Blood kit, Qiagen). *msp-1* and *msp-2* genes were amplified by nested PCR [51], electrophoresed in a 2.5% agarose gel and multiplicity of infection was estimated as the highest number of *msp-1* or *msp-2* alleles detected in each sample.

### RNA extraction and cDNA synthesis

RNA was extracted from snap frozen IE resuspended in 20 volumes of Trizol, using PureLink Micro-to-Midi RNA Purification Kit (Invitrogen). Quantity and integrity of RNA were assessed in Nanodrop spectrophotometer (Thermo Scientific) and 2100 Bioanalyzer (Agilent). Reverse transcription of RNA was performed as previously described [52]. Briefly, RNA was treated with DNAse-I (Invitrogen) for 1 h at 37°C, and complementary DNA (cDNA) was synthesized using Superscript III First Strand Synthesis System (Invitrogen). Controls without reverse transcriptase were processed in parallel.

### Cloning and sequencing

Two regions of DBL2X and DBL3X covering A4 *var2csa* (AY372123) [53] nucleotide positions 2004–2502 (498 bp) and 3819–4365 (546 bp, Figure 1), respectively, were amplified from cDNA with Platinum High-Fidelity Taq Polymerase (Invitrogen). For DBL2X, primers 2XF (5′-CTAATACAATAGGTTTACCCCCAAGAAC-3) and 2XR (5-GGGACCAAATATATAAGAGGTATTCC-3) were used for the primary reaction, and B51 [54] and 2XR for the second amplification. Cycling conditions for both reactions were 94°C for 5 min, 40 cycles of 94°C for 30 s, 50°C for 30 s, 68°C for 1 min and final extension for 5 min at 68°C. DBL3X was amplified using primers 3XF (5-CCAGGTAAAGGAGGCGAGAAA-3) and 3XR (5-CTGCGAGTGATTTATTGAAAGAAAA-3) for the primary PCR. Conditions were set at 94°C for 5 min, 40 cycles of 94°C for 30 s, 45°C for 30 s, 68°C for 1 min, final extension 68°C for 5 min. Primers D3F and D3R1 [42] were used in the nested reaction at an annealing temperature of 50°C. RT negative controls were also processed to discard gDNA contamination. Final products were electrophoresed, purified using Wizard SV Gel and PCR CleanUp system (Promega), ligated into pCR2.1-TOPO vector and transformed into competent One Shot Cells using TOPO-TA cloning Kit (Invitrogen). All colonies with plasmids containing DBL2X and DBL3X inserts were purified and amplified with Platinum High-Fidelity Taq Polymerase using universal primers M13F/M13R. After isopropanol:ethanol precipitation, DNA was sequenced in both senses at Macrogen Inc (Seoul, Korea).

### Sequence analysis

The quality of all chromatograms was revised by eye and sequences were assembled using SeqMan 6.0 (DNASTAR). Mutations were accepted only if they were confirmed in more than one sequence. Nucleotide sequences differing in at least one nucleotide (Genbank accession numbers JQ439999-JQ440365, JQ779990-JQ780047) were entered to BioEdit 7.0.9, translated to amino acids and aligned using ClustalW with default parameters, followed by manual corrections. Multiple alignment of DNA sequences was created in RevTrans 1.4 [55], providing the multiple alignment of amino acid sequences as a template. Identical sequences at nucleotide or amino-acid level were identified using BLASTclust (http://toolkit.tuebingen.mpg.de/blastclust). Shannon entropy at each position of the alignment was calculated in BioEdit and plotted using GraphPad Prism version 5.0. B cell linear epitopes were predicted in BepiPred using a threshold score of 0.9, which has been reported to have a specificity of 91% for epitope prediction [56].

The segmentation analysis was performed as previously described [38], allowing a maximum of 5 sequence types per segment and a maximum segment length of 10 amino acids, since higher maximum lengths (15 or 25 amino acids) did not simplify the segmentation. Sequence types present in ≤2 women were assigned another sequence type if both types diverged in only 1 amino acid (n = 17 out of 17216 total sequence type assignments), or excluded from the analysis if all other types within the segment diverged by 2 or more amino acids (n = 17 out of 17216 sequence type assignments). Likelihood of amino acid substitutions among the most frequent sequences for each sequence type was quantified using the log odd scores from amino acid substitution matrices for extracellular proteins [39].

The difference in the numbers of dS and dN per site was estimated among positions with more than 95% site coverage, using the Nei-Gojobori method with Jukes and Cantor correction [57]. Variance of the difference was calculated using the bootstrap method (500 replicates) and rates of dS and dN were compared by the Z-test of selection using MEGA5 [58]. Tajima's D sliding window analysis and Fu and Li's test were performed in DnaSP 5.0 [59].

### 3D models

The 3D-structure of DBL2X was calculated by submitting the 3D7 sequence (with domain limits defined by Gill *et al.* [60]) to the HHPred server (http://toolkit.tuebingen.mpg.de/hhpred). The structure with highest HHPred score, corresponding to the DBL1α domain of the VarO strain (Protein Data Bank [PDB] 2XU0 [61]), was selected for homology modelling in MODELLER based on the default alignment. The 3D crystal structures of DBL3X were obtained from PDB (3BQK and 3BQI [19]). Molecular graphics were generated in UCSF Chimera version 1.5.3 [62].

### Plasma and peptide ELISA

One hundred plasma samples from pregnant women delivering at the MDH between 2004 to 2005 (collected after obtaining written informed consent) were randomly selected among those who were not infected by *P. falciparum* at the time of delivery [63], to avoid unspecific antibody boosting by placental infection [11]. A pool of plasma samples from hyper-immune Mozambican MG as well as 8 plasmas from non-exposed European pregnant women was included as positive and negative control, respectively. For segments with more than 2 sequence types (S2B, S2C, S3A and S3C) the motif with the highest rate ratio in the association analysis and the reference motif were tested (Table 2). The most frequent sequence in the Mozambican population was selected as representative of each motif for peptide synthesis. Peptides of DBL2X (EYTKDLE, DFTKDLE, EQRQAKVNA, KQRQEKVNA, ECKNKC, ECEKKC) and DBL3X (NLWDKSYG, ELWDKRYG, QNDKKEK, RNPMKEGGEDGKGKQKEGG, NKKQKKNGT, KKQNNKCT) were synthesized at 70% purity by Genscript (Piscataway, USA).

Peptide recognition by plasma was tested by enzyme-linked immunosorbent assay (ELISA). High-binding 96-well microplates (Nunc Maxisorp) were coated overnight at 4°C with 500 ng per well of peptide diluted in carbonate-bicarbonate buffer. Blocking was done with 3% bovine serum albumin (BSA) for 2 h at 37°C and 100 μl of plasma diluted at 1/50 were tested in duplicate. After incubation with peroxidase-conjugated goat anti-human IgG antibodies (Sigma) at a 1/20,000 dilution, the reaction was developed for 15 min with H_2_O_2_ and *o*-phenylenediamine. Specific reactivity of plasmas was obtained as optical density (OD) values measured at 492 nm (Multiskan EX, Labsystems) and normalized by dividing OD of each sample by the OD of the positive control run in each plate.

### Sequences from public databases

VAR2CSA sequences from other geographical areas were obtained from gDNA and cDNA data deposited at Genbank containing the DBL2X or DBL3X regions analysed in the present study. The origin, number of sequences and Genbank accession numbers were: DBL2X: Senegal, *n = *34, GQ358100-GQ358116, GQ358118-GQ358131, GQ358133- GQ358135 [41]; DBL3X: Malawi, *n = *20, DQ286630, DQ286631, DQ306266-DQ306298, AY461586-AY461592, AY461594 [42]; Kenya, *n = *76, JN615483-JN615491, JN615493-JN615506, JN615508-JN615513, JN615515-JN615561 [35]; Senegal, *n = *39, DQ995590-DQ995606, DQ995609-DQ995630 [34]; Papua New Guinea, *n = *48, GQ465375- GQ465385, GQ465387-GQ465390, GQ465393-GQ465396, GQ465398-GQ465425, GQ465427 [43]. The presence and frequency of HDS and LDS was evaluated by searching each HDS and LDS different sequence found in Mozambique (Datasets S1 and S2) in these population sets. Sequences without a perfect match with those from Mozambique were not counted.

### Definitions and statistical methods

Pregnant women were classified as PG (first pregnancy) or MG (at least one previous pregnancy). LBW was defined as newborn's weight at birth <2500 g. Segments in DBL2X and DBL3X were defined as variable if they contained residues with entropy values higher than the median of all positive entropy values for each domain. The number of different DBL2X and DBL3X sequences in each infection was normalized to the number of sequenced clones ([number of different sequences/number of sequenced clones] x [median number of sequenced clones in all samples]) to allow for comparisons between isolates. IgG recognition of peptides tested by ELISA was considered positive if OD in test plasma was higher than mean plus 3 standard deviations of OD in negative controls.

Associations between clinical and parasitological characteristics of pregnant women were evaluated by Mann-Whitney (continuous variables) or Fisher's exact (categorical variables) tests. The association between placental parasite density (dependent variable) and sequence types in a variable segment (independent variable) was evaluated by binomial negative regression including the sequence reads from all clones and a robust variance estimator to account for the effect of repeated measurements within subjects (i.e. each isolate had multiple sequence reads associated to a single parasite density value). For each segment, the sequence type associated with the lowest parasite density value was set as reference. The associations between sequences in each segment and LBW were evaluated by logistic regression and Fisher's exact test, while the associations with parity were analyzed by multinomial logistic regression with parity as independent variable. The global significance in segments with more than two sequence types was estimated by the Wald test. False discovery rate was controlled by Bonferroni correction of the regressions performed for the DBL2X and DBL3X variable segments. McNemar's test was used to compare the antibody recognition between peptides associated with high and low placental *P. falciparum* density. Statistical analyses were conducted in STATA 11.0 (StataCorp).

## Supporting Information

Figure S1 **Tajima's D for DBL2X (** ***A*** **) and DBL3X (** ***B*** **) nucleotide sequence alignments.** Sliding window plot was computed with window lengths of 10 sites and a step size of 5 sites. The location of segments containing signatures of high parasite density is indicated.
(PDF)

Table S1 Number of isolates and most frequent amino acid sequences in segments associated with *P. falciparum* density in the placenta. Results of association between sequence types, parity and low birth weight (LBW) are listed.
(DOCX)

Dataset S1 **Multiple alignment of DBL2X sequences.** Alignment of 176 unique amino acid sequences transcribed by 20 *P. falciparum* placental isolates. Segments containing signatures of high parasite density are colored.
(XLSX)

Dataset S2 **Multiple alignment of DBL3X sequences.** Alignment of 191 unique amino acid sequences transcribed by 20 *P. falciparum* placental isolates. Segments containing signatures of high parasite density are colored.
(XLSX)
