# Supplementary figures and images for: VAR2CSA Signatures of High Plasmodium falciparum Parasitemia in the Placenta
Source: PLoS One. 2013 Jul 25;8(7):e69753. doi: 10.1371/journal.pone.0069753 (PMC3723727; doi:10.1371/journal.pone.0069753)

**A.**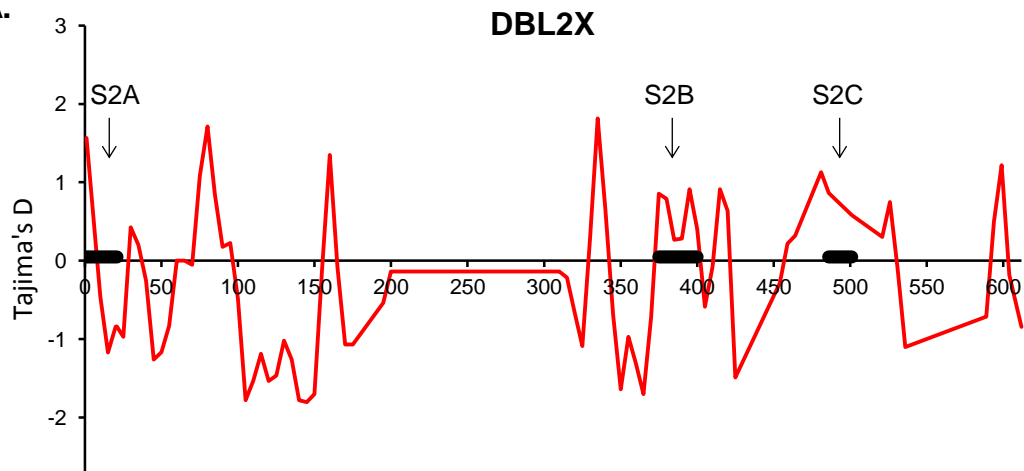**B.**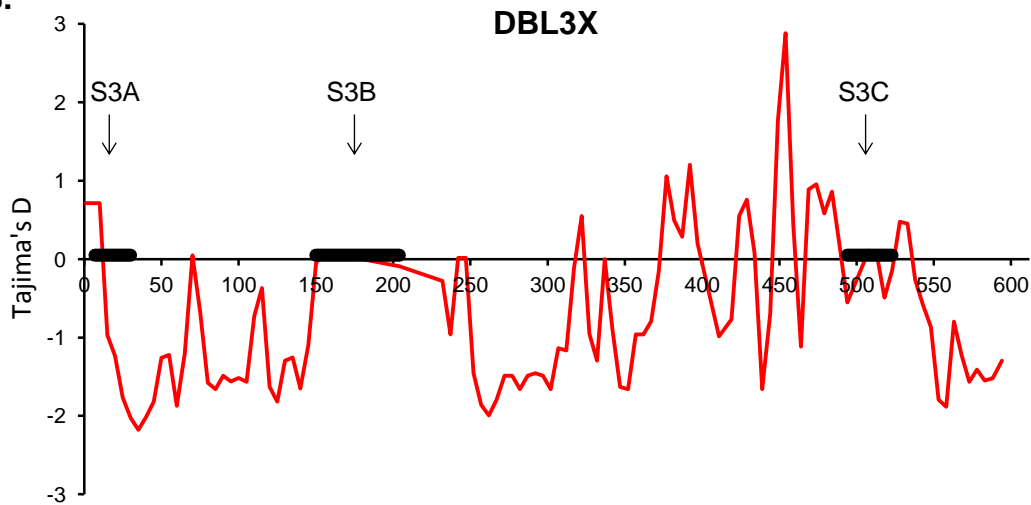

Supplement: Figure S1 — Tajima's D for DBL2X ( A ) and DBL3X ( B ) nucleotide sequence alignments. Sliding window plot was computed with window lengths of 10 sites and a step size of 5 sites. The location of segments containing signatures of high parasite density is indicated. (PDF) [file pone.0069753.s001.pdf]
