# Supplementary material for: VAR2CSA Signatures of High Plasmodium falciparum Parasitemia in the Placenta
Source: PLoS One. 2013 Jul 25;8(7):e69753. doi: 10.1371/journal.pone.0069753 (PMC3723727; doi:10.1371/journal.pone.0069753)
Supplement: Table S1 — Number of isolates and most frequent amino acid sequences in segments associated with P. falciparum density in the placenta. Results of association between sequence types, parity and low birth weight (LBW) are listed. (DOCX) [file pone.0069753.s002.docx]

| **Sequence type** | **Isolates, n (%, out of 20)** | **Clones, N** | **Most frequent sequence^a^ and clones, n (%)** | | **Parity**^b^ | | **LBW**^c^ | |
| --- | --- | --- | --- | --- | --- | --- | --- | --- |
|  |  |  |  |  | **RRR (95% CI)** | ***P*** | **OR (95% CI)** | ***P*** |
|  |  |  |  |  |  |  |  |  |
| **DBL2X** |  |  |  |  |  |  |  |  |
|  |  |  |  |  |  |  |  |  |
| S2A.1 (HDS) | 16 (80) | 232 | EYTKDLE | 229 (99) | 0.74 (0.1, 4.4) | 0.731 | 3.1 (0.4, 22.9) | 0.271 |
| S2A.2 (LDS) | 13 (65) | 141 | DFTKDLE | 116 (82) | *ref.* |  | *ref.* |  |
|  |  |  |  |  |  |  |  |  |
| S2B.1 (HDS) | 9 (45) | 110 | EQRQAKVNA | 81 (74) | 1.0 (0.1, 10.2) | 0.970 | 0.1 (0.01, 1.6) | 0.426 |
| S2B.2 (HDS) | 5 (25) | 58 | KQRQENVNA | 56 (97) | 4.1 (0.3, 59.0) | 0.298 | 0.4 (0.03, 4.2) | 0.112 |
| S2B.3 | 10 (50) | 128 | KQRQEKVKP | 34 (27) | 2.5 (0.2, 25.6) | 0.433 | 0.1 (0.01, 1.8) | 0.122 |
| S2B.4 (LDS) | 9 (45) | 77 | KQRQEKVNA | 35 (45) | *ref.* |  | *ref.* |  |
|  |  |  |  |  |  |  |  |  |
| S2C.1 (HDS) | 12 (60) | 108 | ECKNKC | 82 (76) | 35.9 (3.1, 419.0) | 0.004 | ^d^ *(see Footnote)* |  |
| S2C.2 (HDS) | 16 (80) | 232 | K----C | 200 (86) | 42.8 (4.6, 396.8) | 0.001 |  |  |
| S2C.3 (LDS) | 3 (15) | 32 | ECEKKC | 31 (97) | *ref.* |  |  |  |
|  |  |  |  |  |  |  |  |  |
| **DBL3X** |  |  |  |  |  |  |  |  |
|  |  |  |  |  |  |  |  |  |
| S3A.1 (HDS) | 11 (55) | 105 | NLWDKSYG | 87 (83) | 2.5 (0.3, 23.7) | 0.412 | 0.7 (0.1, 7.2) | 0.737 |
| S3A.2 (HDS) | 13 (65) | 150 | ELWYKSYG | 114 (76) | 1.3 (0.2, 9.5) | 0.772 | 1.2 (0.1, 12.3) | 0.904 |
| S3A.3 (LDS) | 9 (45) | 71 | ELWDKRYG | 33 (46) | *ref.* |  | *ref.* |  |
|  |  |  |  |  |  |  |  |  |
| S3B.1 (HDS) | 20 (100) | 292 | QN-------DKK----EK- | 100 (34) | 12.6 (1.3, 126.5) | 0.005 | ^d^ *(see Footnote)* |  |
| S3B.2 (LDS) | 5 (25) | 36 | RNPMKEGGEDGKGKQKEGG | 34 (94) | *ref.* |  |  |  |
|  |  |  |  |  |  |  |  |  |
| S3C.1 (HDS) | 14 (70) | 171 | NKKQKKNGT | 108 (63) | 1.3 (0.2, 7.9) | 0.786 | 3.1 (0.7, 14.4) | 0.146 |
| S3C.2 | 8 (40) | 63 | NKKKKKNGT | 34 (54) | 1.7 (0.2, 15.8) | 0.647 | 9.9 (1.0, 101.7) | 0.053 |
| S3C.3 (LDS) | 10 (50) | 76 | -KKQNNKCT | 46 (61) | *ref.* |  | *ref.* |  |

**Table S1.** Number of isolates and most frequent amino acid sequences in segments associated with *P. falciparum* density in the placenta. Results of association between sequence types, parity and low birth weight (LBW) are listed.

HDS, high (placental parasite) density signature; LDS, low (placental parasite) density signature; RRR, relative risk ratio (i.e., relative risk ratio for being PG when carrying that sequence type as compared to reference sequence type); CI, confidence interval; OR, odds ratio (i.e., odds for LBW if carrying that sequence type as compared to reference sequence type); *ref.,* reference category (set as the sequence type associated with the lowest parasite density value).

^a^ Displayed as aligned sequences. Colored amino acids indicate favorable (green) or unfavorable (red) exchanges (see *Materials and Methods)*. For S3B, a conserved lysine residue (K1328 in A4 strain) is underlined.

^b^Multinomial logistic regression with robust estimation of variance. None of the segments contained a significant association with parity after Bonferroni correction of Wald tests (thresholds: *P*[DBL2X]=0.0028 and *P*[DBL3X]=0.0036): *P*[S2A]=0.731, *P*[S2B]=0.591, *P*[S2C]=0.0045, *P*[S3A]=0.686, *P*[S3B]=0.005, *P*[S3C]=0.900.

^c^ Logistic regression with robust estimation of variance. None of the segments contained a significant association with LBW after Bonferroni correction of Wald tests (thresholds: *P*[DBL2X]=0.0028 and *P*[DBL3X]=0.0036): *P*[S2A]=0.271, *P*[S2B]=0.315, *P*[S3A]=0.832, *P*[S3C]=0.122.

^d^ Logistic regression models cannot be applied since no deliveries with LBW were observed among women infected with parasites carrying S2C.3 (isolates from 3 women, 32 sequences) or S3B.2 (isolates from 5 women, 36 sequences). Alternatively, Fisher’s exact test for S2C: number of S2C.1 clones in women delivering LBW babies=38(35%), number of S2C.2 clones in women delivering LBW babies=57(25%), *P*<0.001. Fisher’s exact test for S3B: number of S3B.1 clones in women delivering LBW babies=70(24%), *P*<0.001.
